# Improved Simultaneous Multi-slice imaging with Composition of k-space Interpolations (SMS-COOKIE) for myocardial T_1_ mapping

**DOI:** 10.1371/journal.pone.0283972

**Published:** 2023-07-21

**Authors:** Ömer Burak Demirel, Sebastian Weingärtner, Steen Moeller, Mehmet Akçakaya

**Affiliations:** 1 Electrical and Computer Engineering, University of Minnesota, Minneapolis, Minnesota, United States of America; 2 Center for Magnetic Resonance Research, University of Minnesota, Minneapolis, Minnesota, United States of America; 3 Department of Imaging Physics, Delft University of Technology, Delft, The Netherlands; University of Bologna, ITALY

## Abstract

The aim of this study is to develop and evaluate a regularized Simultaneous Multi-Slice (SMS) reconstruction method for improved Cardiac Magnetic Resonance Imaging (CMR). The proposed reconstruction method, SMS with COmpOsition of k-space IntErpolations (SMS-COOKIE) combines the advantages of Iterative Self-consistent Parallel Imaging Reconstruction (SPIRiT) and split slice-Generalized Autocalibrating Partially Parallel Acquisitions (GRAPPA), while allowing regularization for further noise reduction. The proposed SMS-COOKIE was implemented with and without regularization, and validated using a Saturation Pulse-Prepared Heart rate Independent inversion REcovery (SAPPHIRE) myocardial T_1_ mapping sequence. The performance of the proposed reconstruction method was compared to ReadOut (RO)–SENSE-GRAPPA and split slice-GRAPPA, on both retrospectively and prospectively three-fold SMS-accelerated data with an additional two-fold in-plane acceleration. All SMS reconstruction methods yielded similar T_1_ values compared to single band imaging. SMS-COOKIE showed lower spatial variability in myocardial T_1_ with significant improvement over RO-SENSE-GRAPPA and split slice-GRAPPA (*P* < 10^−4^). The proposed method with additional locally low rank (LLR) regularization reduced the spatial variability, again with significant improvement over RO-SENSE-GRAPPA and split slice-GRAPPA (*P* < 10^−4^). In conclusion, improved reconstruction quality was achieved with the proposed SMS-COOKIE, which also provided lower spatial variability with significant improvement over split slice-GRAPPA.

## Introduction

Quantitative Cardiac Magnetic Resonance Imaging (CMR) has received substantial interest in the assessment of pathological variations in the myocardium [1]. Specifically, myocardial tissue characterization with quantification of different relaxation parameters, such as T_1_,T_2_,T_1*ρ*_ and ${\text{T}}_{2}^{*}$, has emerged as one of the main quantitative CMR applications [2], showing great promise in the assessment of various cardiomyopathies [3–6]. In particular, T_1_ mapping, a pixel-wise parametric map of spin-lattice relaxation time, has shown promise in acute and chronic myocardial infraction [7–9], acute ischemia and inflammation [9–11], aortic stenosis [12] and cardiac amyloidosis [13, 14], among others.

In myocardial T_1_ mapping, multiple images of a slice are acquired with different T_1_ weightings, and pixel-wise quantification is performed using a parametric model [15]. In clinical applications, three slice coverage of the myocardium is recommended [16] and each slice is typically acquired in a separate breath-hold [15]. The scan time increases with multiple breath-holds due to the rest periods between each breath-held acquisition. Additionally, multiple breath-holds are a significant source of patient discomfort especially for elderly patients or patients with dyspnea.

Simultaneous multi-slice (SMS) or multi-band (MB) imaging has been proposed as an accelerated imaging technique to improve the coverage of multi-slice imaging without increasing scan time. In SMS imaging, multiple slices are excited with multi-frequency excitation pulses [17]. Since acceleration is achieved by acquiring multiple slices at the same time there is no inherent SNR loss compared to 2D single-slice imaging except for noise amplification based on the coil geometry [18]. Coupled with Controlled Aliasing in Parallel Imaging Results in Higher Acceleration (CAIPIRINHA) [19] to further promote dissimilarity between coil profiles in the acquired slices, SMS imaging provides improved coverage with minimal SNR loss. Thus, SMS imaging has gained interest as a strategy for CMR applications [20–27], including myocardial T_1_ mapping [23, 27]. In this context, slice Generalized Autocalibrating Partially Parallel Acquisitions (Slice GRAPPA) reconstruction was shown to lower cross-talk between simultaneously acquired slices, referred to as inter-slice leakage [28, 29], compared to Sensitivity Encoding (SENSE)-type reconstructions, and to lead to comparable accuracy with respect to single band imaging at the expense of reduced precision [23]. However, only linear reconstruction algorithms, which do not employ regularization, were used in [23], leading to reduced precision compared with single band imaging.

In this work, we propose an alternative SMS imaging reconstruction technique, SMS with *CO*mp*O*sition of *k*-space *I*nt*E*rpolations (SMS-COOKIE) that combines the advantages of different k-space interpolation techniques, while enabling further reduction in noise by additional regularization. The proposed technique is compared to existing techniques for SMS reconstruction in myocardial T_1_ mapping, and is shown to improve precision without compromising accuracy.

## Methods

### Conventional reconstruction methods

#### GRAPPA and split slice-GRAPPA

GRAPPA is a k-space interpolation technique for parallel imaging reconstruction [30]. GRAPPA estimates the missing k-space points in a uniformly sub-sampled k-space using linear shift-invariant convolution kernels. The weights of the convolution kernels are calibrated either from a separate reference scan or the fully sampled center of the k-space which is also referred to as autocalibration signal (ACS).

GRAPPA-like reconstruction methods have also been proposed for SMS imaging [29, 31–33]. While earlier works utilized k-space interpolation akin to GRAPPA, later works relied on a projection-type approach [29, 31]. Slice GRAPPA estimated separate k-spaces for each slice using slice-specific sets of GRAPPA convolutional kernels on the acquired SMS data [31]. However, this approach estimated a projection from the SMS data for each individual slice with no constraints on the other slices, and thus was inherently susceptible to inter-slice leakage [29]. Later, split slice-GRAPPA was proposed [29] with an additional constraint during calibration for leakage-blocking. To this end, split slice-GRAPPA enforces the rest of the slices to zero during a slice-specific weight calibration to prevent inter-slice leakage artifacts. Following calibration, split slice-GRAPPA performs the following projection-type equation reconstruction:

$$\begin{array}{c} G_{SMS}\kappa_{SMS}={\left[\kappa_{1}\;\cdots \;\kappa_{n}\right]}^{T}, \end{array}$$ (1)

where ***κ***_*SMS*_ is the acquired SMS k-space data, **G**_*SMS*_ is the split slice-GRAPPA convolution operator and ***κ***_*i*_ is the k-space data of the *i*^*th*^ slice and *n* is the total number of slices. By design, split slice-GRAPPA is effective in removing inter-slice residual aliasing artifacts, but it is prone to noise amplification, which can be detrimental at high acceleration rates [34]. Additionally, for higher acceleration rates, SMS imaging may be combined with in-plane acceleration. In this case, a two-stage reconstruction [35] is typically applied, starting with split slice-GRAPPA [29] for multi-slice unaliasing, followed by in-plane GRAPPA [30]. The first step of applying split slice-GRAPPA leads to disentangled slice with in-plane aliasing. Subsequently, standard GRAPPA reconstruction is applied to interpolate the missing points along the phase encode direction.

#### SPIRiT

Iterative Self-consistent Parallel Imaging Reconstruction (SPIRiT) is an alternative parallel imaging reconstruction technique that performs k-space interpolation to estimate missing k-space points and can work with arbitrary sub-sampling patterns [36]. SPIRiT reconstruction solves the following objective function:

$$\begin{array}{c} \text{arg}\;\underset{\kappa }{\text{min}}{\left\|P_{\Omega }\kappa -y\right\|}_{2}^{2}+{\left\|G\kappa -\kappa \right\|}_{2}^{2}, \end{array}$$ (2)

where **P**_Ω_ is the sub-sampling operator that only picks the acquired k-space data specified by Ω, ***κ*** is the k-space data across all coils, **y** is the acquired k-space data across all coils and **G** is the SPIRiT convolution operator. SPIRiT jointly enforces consistency with the acquired data (first term in Eq 2) and coil self-consistency from multiple coils (second term in Eq 2). SPIRiT works with arbitrary sampling patterns and it allows additional regularization. SPiRiT-type reconstruction has also been employed in non-Cartesian SMS imaging [26, 37]. The main advantage of the SPIRiT formulation for SMS is that it enables the incorporation of regularization terms and also allows reconstruction from arbitrary undersampling patterns, though it may suffer from residual aliasing at high acceleration rates [38, 39].

### SMS with Composition of K-space Interpolations (SMS-COOKIE)

**Fig 1:**
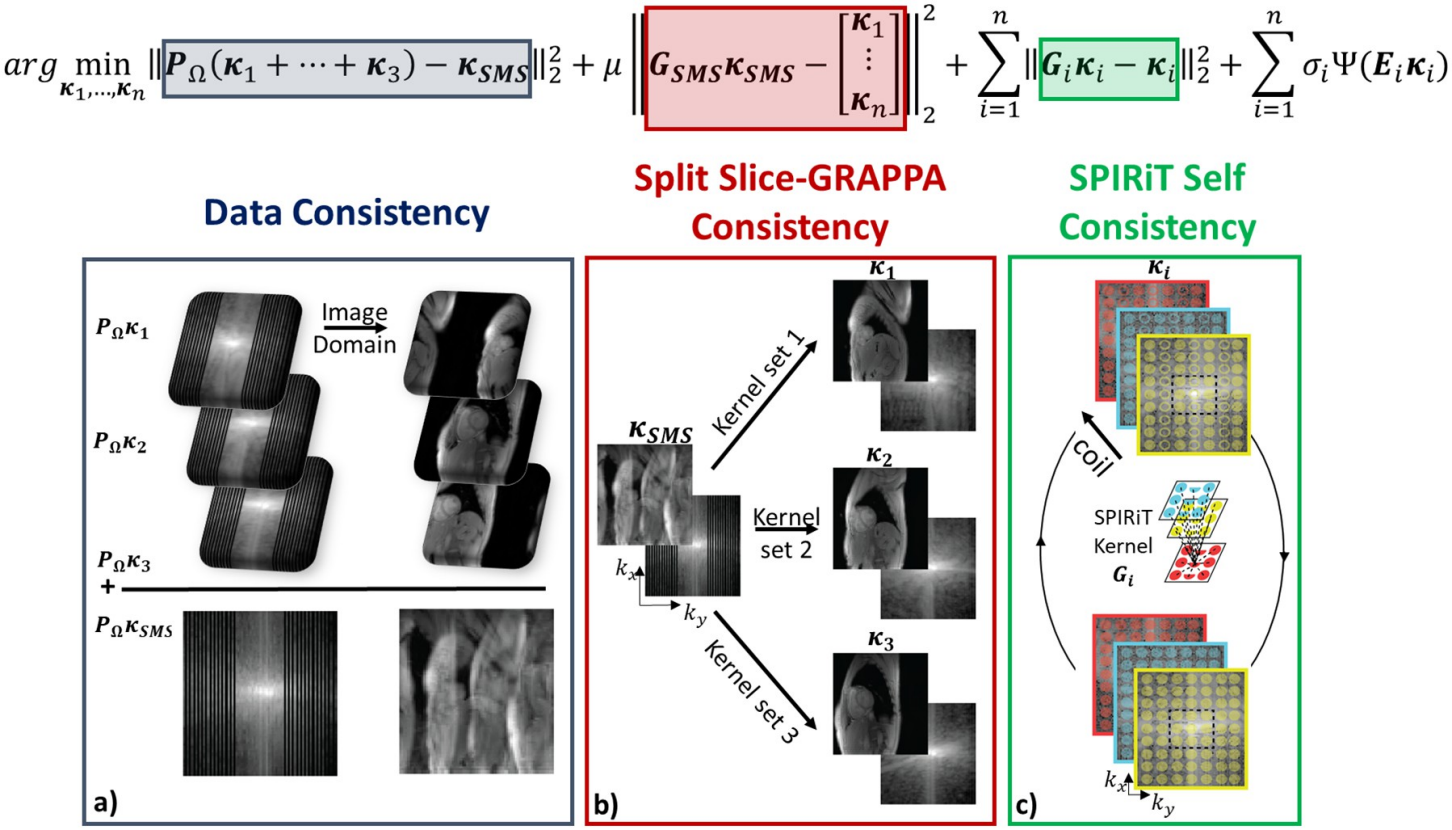
A schematic of the SMS-COOKIE objective function in Eq 3. (a) Data consistency term (depicted with blue box) enforces consistency with the acquired k-space data ***κ***_*SMS*_, (b) Split slice-GRAPPA consistency term (depicted with maroon box) provides noisy but reliable estimates of individual k-space slices, (c) SPIRiT term (depicted with green box) further enforces coil self-consistency and improves the individual k-space estimations. An SMS acceleration factor of *n* = 3 is shown and regularization terms are not depicted.

In this work, we sought to use the advantages of the two aforementioned k-space reconstruction strategies. SMS-COOKIE incorporates both the aliasing artifact reduction performance of split slice-GRAPPA and the regularization benefits of SPIRiT. To this end, the following objective function is solved:

$$\begin{array}{c} \text{arg}\;\underset{\left\{\kappa_{1},\cdots ,\kappa_{n}\right\}}{\text{min}}{\left\|P_{\Omega }(\kappa_{1}+\cdots +\kappa_{n})-\kappa_{SMS}\right\|}_{2}^{2}+\mu {\left\|G_{SMS}\kappa_{SMS}-{\left[\kappa_{1}\;\cdots \;\kappa_{n}\right]}^{T}\right\|}_{2}^{2}\\ +\sum_{i=1}^{n}{\left\|G_{i}\kappa_{i}-\kappa_{i}\right\|}_{2}^{2}+\sum_{i=1}^{n}\sigma_{i}\Psi (E_{i}\kappa_{i}) \end{array}$$ (3)

where **P**_Ω_ is the sub-sampling operator, ***κ***_*SMS*_ is the acquired SMS data, ***κ***_*i*_ is the k-space data of the *i*^*th*^ slice, **G**_*i*_ is the SPIRiT convolution operator of the *i*^*th*^ slice, Ψ is the regularizer, **E**_*i*_ is the SENSE-1 operator, **G**_*SMS*_ is the split slice-GRAPPA operator from Eq 1, *μ* and *σ*_*i*_ are the weight terms and *n* is the total number of slices. Here the SENSE-1 operator (**E**_*i*_) applies the inverse Fourier transform of all the channels of the *i*^*th*^ slice k-space and combines them into one image using coil sensitivity maps [18, 40]. Fig 1 shows a schematic description of data consistency (first term), split slice-GRAPPA consistency (second term) and SPIRIT coil self-consistency (third term) of the proposed SMS-COOKIE method.

The objective function in Eq 3 was solved using Alternating Direction Method of Multipliers (ADMM) [41] with the following three sub-problems. The first sub-problem is solved with respect to main variables {***κ***_1_, …, ***κ***_*n*_} at iteration *t*:

$$\begin{array}{cc} \{\kappa_{1}^{\left(t\right)},\cdots ,\kappa_{n}^{\left(t\right)}\}=\text{arg} & \underset{\left\{\kappa_{1},\cdots ,\kappa_{n}\right\}}{\text{min}}{\left\|P_{\Omega }(\kappa_{1}+\cdots +\kappa_{n})-\kappa_{SMS}\right\|}_{2}^{2}\\ & +\mu {\left\|G_{SMS}\kappa_{SMS}-{\left[\kappa_{1}\;\cdots \;\kappa_{n}\right]}^{T}\right\|}_{2}^{2}\\ & +\sum_{i=1}^{n}{\left\|G_{i}\kappa_{i}-\kappa_{i}\right\|}_{2}^{2}+\sum_{i=1}^{n}\frac{\rho }{2}{\left\|E_{i}\kappa_{i}-z_{i}^{\left(t-1\right)}+\frac{\lambda_{i}^{\left(t-1\right)}}{\rho }\right\|}_{2}^{2}, \end{array}$$ (4)

where ${\left\{z_{i}\right\}}_{i=1}^{n}$ are introduced as auxiliary variables for constraining ${\left\{E_{i}\kappa_{i}\right\}}_{i=1}^{n}$, ${\left\{z_{i}^{\left(t-1\right)}\right\}}_{i=1}^{n}$ are the (*t* − 1)^*th*^ iteration of these auxiliary variables and ${\left\{\lambda_{i}\right\}}_{i=1}^{n}$ are the dual variables. The update for {**z**_1_, ⋯, **z**_*n*_} are given as:

$$\begin{array}{c} \left\{z_{1}^{\left(t\right)},\cdots ,z_{n}^{\left(t\right)}\right\}=\text{arg}\underset{\left\{z_{1},\cdots ,z_{n}\right\}}{\text{min}}{\left\|E_{i}\kappa_{i}^{\left(t\right)}-z_{i}+\frac{\lambda_{i}^{\left(t-1\right)}}{\rho }\right\|}_{2}^{2}+\frac{\sigma_{i}}{\rho }\Psi (z_{i}), \end{array}$$ (5)

where ${\left\{\lambda_{i}^{\left(t-1\right)}\right\}}_{i=1}^{n}$ are the (*t* − 1)^*th*^ iteration of the dual variables. Finally, the dual variables are updated as:

$$\begin{array}{c} \lambda_{i}^{\left(t\right)}=\lambda_{i}^{\left(t-1\right)}+\rho (E_{i}\kappa_{i}^{\left(t\right)}-z_{i}^{\left(t\right)}). \end{array}$$ (6)

### In vivo imaging

Imaging was performed on a 3T Siemens Magnetom Prisma (Siemens Healthineers, Erlangen, Germany) in 6 healthy subjects (3 men, 3 women, mean age: 36±16 years) with no contraindications to MRI. This study was approved by our institutional review board, and written informed consent was obtained before each examination.

Myocardial T_1_ mapping was performed using an electrocardiogram (ECG)-triggered SAturation Pulse-Prepared Heart rate independent Inversion REcovery (SAPPHIRE) sequence [42] with Fast Low Angle Shot (FLASH) imaging. Single band SAPPHIRE was performed in three breath-holds to cover three slices, whereas SMS SAPPHIRE was performed with a single breath-hold to simultaneously cover three slices [23]. In addition to SMS excitation, further in-plane acceleration of rate 2 was utilized. Fifteen linearly distributed inversion times were utilized between minimum inversion time of 185 ms and maximum inversion time determined by the start of diastolic phase to acquire 15 images with different T_1_ weightings. To reduce the noise amplification, CAIPIRINHA was utilized with a phase increment of 2*π*/3 that provides 1/3 FOV shifts in between the adjacent slices [19].

The relevant imaging parameters were [23]: Repetition time (TR)/ echo time (TE)/ flip angle (FA) = 3.6 ms/1.8 ms/10°; FOV = 320×320 mm^2^; spatial resolution = 2×2.1 mm^2^; slice thickness = 10 mm; bandwidth = 505 Hz/pixel; linear k-space ordering with uniform in-plane undersampling = 2, 24 central lines and partial Fourier = 6/8. A separate free-breathing scan without ECG-triggering corresponding to 64 reference lines was acquired as calibration data for SMS reconstruction with identical imaging parameters but low spatial resolution = 2×5 mm^2^.

### Reconstruction experiments

The acquired raw data of three single band slices for T_1_ mapping were processed offline in MATLAB (MathWorks, Natick, Massachusetts, USA). A retrospective 3-fold SMS and 2-fold in-plane acceleration with 24 ACS lines was performed to evaluate the performance of the proposed SMS-COOKIE and state of the art methods with respect to a ground truth. 2*π*/3 CAIPIRINHA shifts were performed to provide 1/3 FOV shifts between the adjacent slices.

ReadOut (RO)–SENSE-GRAPPA and split slice-GRAPPA were compared to proposed SMS-COOKIE with and without regularization. 6 × 6 kernel size was used in RO-SENSE-GRAPPA kernels, which were calibrated on low-resolution reference data [33]. Multi-slice aliasing with 5 × 5 kernel size was used in in split slice-GRAPPA, followed by in-plane GRAPPA with 5 × 4 kernels. SPiRiT kernels in SMS-COOKIE were calibrated using 7 × 7 kernel size. The split slice-GRAPPA weight term in Eq 3, *μ* was empirically tuned in a subject to 7.5 × 10^−3^ (S1 Fig in S1 File). All kernels for RO-SENSE-GRAPPA, split slice-GRAPPA (both SMS and in-plane) and SPiRiT kernels in SMS-COOKIE were calibrated on separate reference scans with 64 phase encode lines. Regularization was incorporated into SMS-COOKIE using a locally low-rank (LLR) [43–45] constraint as follows:

$$\begin{array}{c} \Psi \left(x\right)=\sum_{k}{\left\|B_{k}^{b}\left(x\right)\right\|}_{*}, \end{array}$$ (7)

where a *b* × *b* block is extracted by the $B_{k}^{b}$ operator whose top-left corner is at pixel *k* and || ⋅ ||_*_ is the nuclear norm. These blocks are vectorized and stacked up into a *b*^2^×*n*_*T*_ matrix, where *n*_*T*_ = 15 was the number of T_1_-weighted images in the series. *b* = 8 was employed as the block size in this study. Eq 5 was solved using singular value thresholding by setting the thresholding parameter *σ*_*i*_/*ρ* to 0.08 times the *ℓ*_∞_ norm of the SENSE-1 image for the corresponding slice in regularized SMS-COOKIE. In regularized cases, the thresholding parameter was empirically set using an additional subject (S1 Fig in S1 File).

Additionally, prospectively 3-fold SMS-accelerated raw data for T_1_ mapping with 2-fold in-plane acceleration were reconstructed with RO-SENSE-GRAPPA, split slice-GRAPPA and proposed SMS-COOKIE. RO–SENSE-GRAPPA, split slice-GRAPPA and SMS-COOKIE used the same kernel sizes as in the retrospectively SMS-accelerated study. The thresholding parameter for regularized SMS-COOKIE was divided by 3 with the prospective acceleration to account for the higher SNR of the prospectively SMS-accelerated data.

### Image and data analysis

Image quality assessment of the reconstructions was first performed using the retrospectively SMS-accelerated single band acquisitions. The quality of the reconstructions was evaluated using peak signal-to-noise ratio (PSNR) and structural similarity index (SSIM) across all subjects, all slices and all *T*_1_ weighted images. The error images were calculated as the difference between reconstructed slices and the reference single band acquisitions. In addition to these quantitative image quality metrics, inter slice leakage artifacts, defined as the leakage between unaliased simultaneously acquired slices or cross talk between the slices [29, 46], were also assessed. Specifically, leakage analysis was performed for all linear methods by picking a slice of interest among the three slices, performing the reconstruction based on the data from this slice, and quantifying the residual signal in the two non-input slices as the leakage [23]. Finally, g-factor analysis was performed for all linear methods using Monte-Carlo-based simulations [47] with 256 different random instances.

Following the analyses on retrospectively SMS-accelerated data, quantitative T_1_ maps were generated for each of the three slices using a 3-parameter fit [23], for both retrospectively SMS-accelerated and prospectively SMS-accelerated datasets. Manually drawn regions were utilized to quantitatively analyze 16 AHA segments of the myocardium [48]. To evaluate the performance of the reconstruction methods, two assessment techniques were employed. First, T_1_ values (ms) were estimated as the mean value in the region of interest (ROI) to evaluate the accuracy. Second, spatial variability of the T_1_ maps was computed as the standard deviation in the ROI as a surrogate for the precision. Spatial variability as defined by regional standard deviation in the quantitative maps was considered a proxy for noise-resilience of the evaluated reconstruction methods [23]. In all cases, T_1_ values and spatial variability were reported as mean ± standard deviation, calculated across all subjects. Statistical difference in T_1_ values and spatial variability was assessed using Kruskal-Wallis group test with Bonferroni correction. A *P*-value <.05 was considered significant.

### Numerical phantom experiments

MRXCAT cardiovascular MRI numerical phantom [49] was used to evaluate the performance of different reconstruction techniques. To match the in-vivo study, a 3-fold SMS and 2-fold in-plane acceleration with 24 central lines was simulated. Additionally, a 4-fold SMS without in-plane acceleration and 2-fold SMS with 3-fold in-plane accelerations by keeping 24 central lines were simulated for further performance evaluation and are provided in S1 File. 1/SMS FOV shifts were applied between the adjacent excited slices using CAIPIRINHA [19], as in the in vivo study. The numerical phantom imaging parameters were matched to the in vivo imaging in terms of FOV, inversion times, SNR, number of coils and the distance of the coils from the center of origin. Myocardium and blood compartments were simulated with *T*_1_ = 1500 ms and *T*_1_ = 2200 ms, respectively, each with a uniformly random variability of 150ms., based on previously reported values with SAPPHIRE acquisitions in healthy subjects at 3T [50]. Reconstruction techniques were used with the details given in the Reconstruction Experiments subsection. Peak signal-to-noise ratio (PSNR) and structural similarity index measure (SSIM) were calculated between reference images and all reconstruction techniques.Quantitative fitting and statistical analyses of the *T*_1_ maps were done as described in the **Image and Data Analysis** subsection.

## Results

### Retrospectively SMS-accelerated myocardial T_1_ mapping results

**Fig 2:**
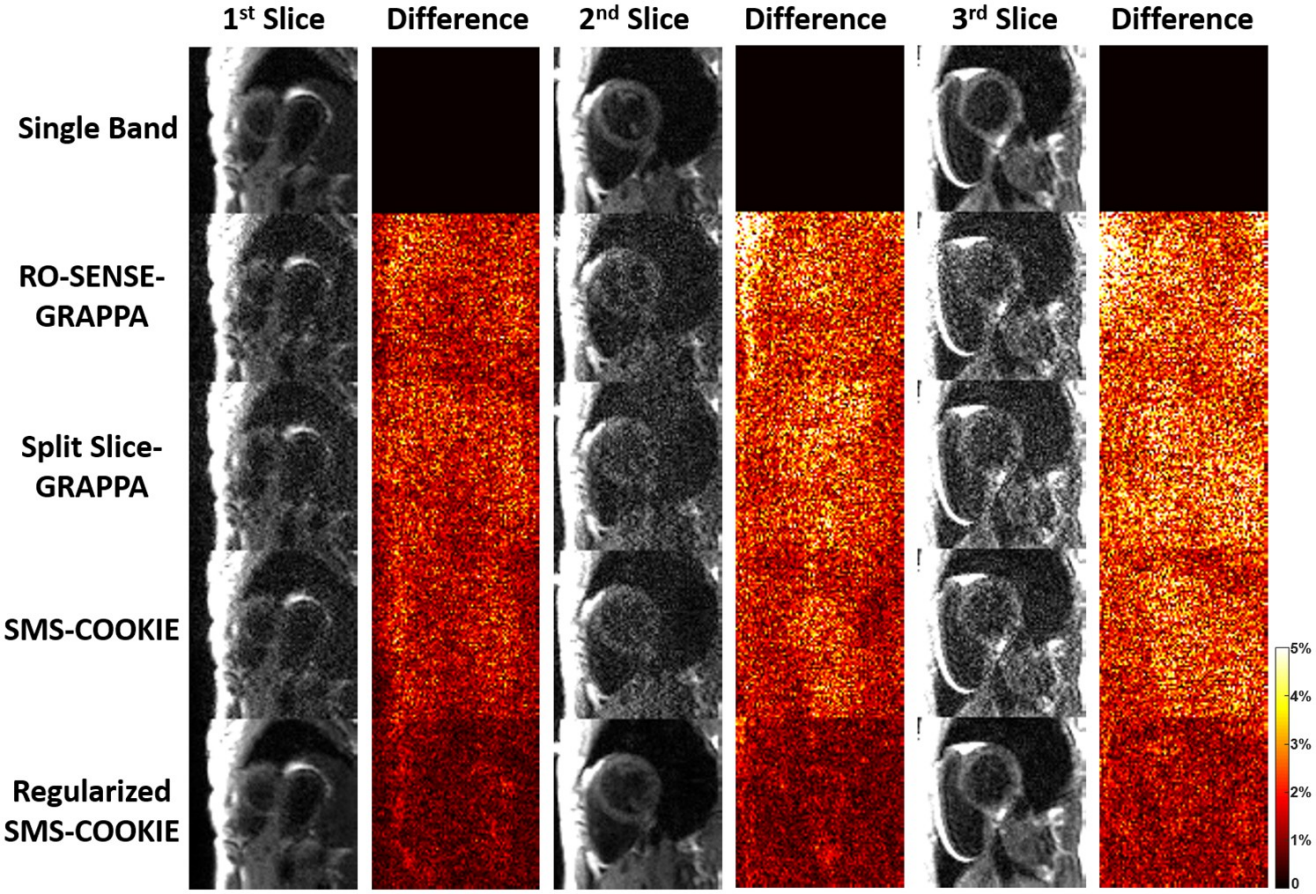
Representative T_1_ weighted images with low SNR from a retrospectively SMS-accelerated dataset, reconstructed using RO-SENSE-GRAPPA, split slice-GRAPPA, proposed SMS-COOKIE and proposed regularized SMS-COOKIE. Single band images are shown in the top row as reference, and difference images are obtained by subtracting them from the reconstructions. Regularized SMS-COOKIE shows the lowest error and visually similar results compared to single band reference images.

Fig 2 shows representative results from retrospectively three-fold SMS and two-fold in-plane accelerated images. T_1_-weighted images with low SNR are shown, where the single band acquisitions are depicted in the top row as a reference. The differences to single band images are shown next to each slice. For this subject, RO-SENSE-GRAPPA shows the lowest PSNR and SSIM metrics (34.7 dB, 80.3%) followed by split slice-GRAPPA with slightly improved PSNR metrics (35.1 dB, 82.3%). SMS-COOKIE improves upon both these methods (38.3 dB, 87.4%), and regularized SMS-COOKIE shows the highest PSNR and SSIM metrics (40.7 dB, 88.1%). The error images depicted next to the reconstructed images are in line with these observations, where regularized SMS-COOKIE shows the lowest difference compared to single band references.

Table 1 depicts the PSNR and SSIM metrics averaged over all subjects, 15 images and three slices of each reconstruction method. RO-SENSE-GRAPPA shows the lowest PSNR and SSIM metrics, where split slice-GRAPPA improves only 2.1% in terms of PSNR and 1.16% in terms of SSIM. SMS-COOKIE improves upon split slice-GRAPPA by 6.9% in terms of PSNR and by 7.57% in terms of SSIM. Regularized SMS-COOKIE shows the highest PSNR and SSIM metrics compared to all other methods. Regularized SMS-COOKIE improves upon SMS-COOKIE without regularization by 10.3% in terms of PSNR and 9.5% in terms of SSIM.

**Table 1:** Average PSNR and SSIM metrics over all subject, 15 images and all three slices. Regularized SMS-COOKIE shows the highest PSNR performance with 23.2%, 20.5% and 10.3% improvement compared to RO-SENSE-GRAPPA, split slice-GRAPPA and SMS-COOKIE. Likewise, regularized SMS-COOKIE shows the highest SSIM among all methods, with 21.1%, 19.7% and 9.5% improvement compared to RO-SENSE-GRAPPA, split slice-GRAPPA and SMS-COOKIE.

Method	PSNR	SSIM
RO-SENSE-GRAPPA	31.5 ± 3.8	76.4 ± 12.4
Split Slice-GRAPPA	32.2 ± 3.9	77.3 ± 12.5
SMS-COOKIE	34.6 ± 4.5	83.7 ± 11.1
Regularized SMS-COOKIE	38.8 ± 3.9	92.5 ± 5.9

**Fig 3:**
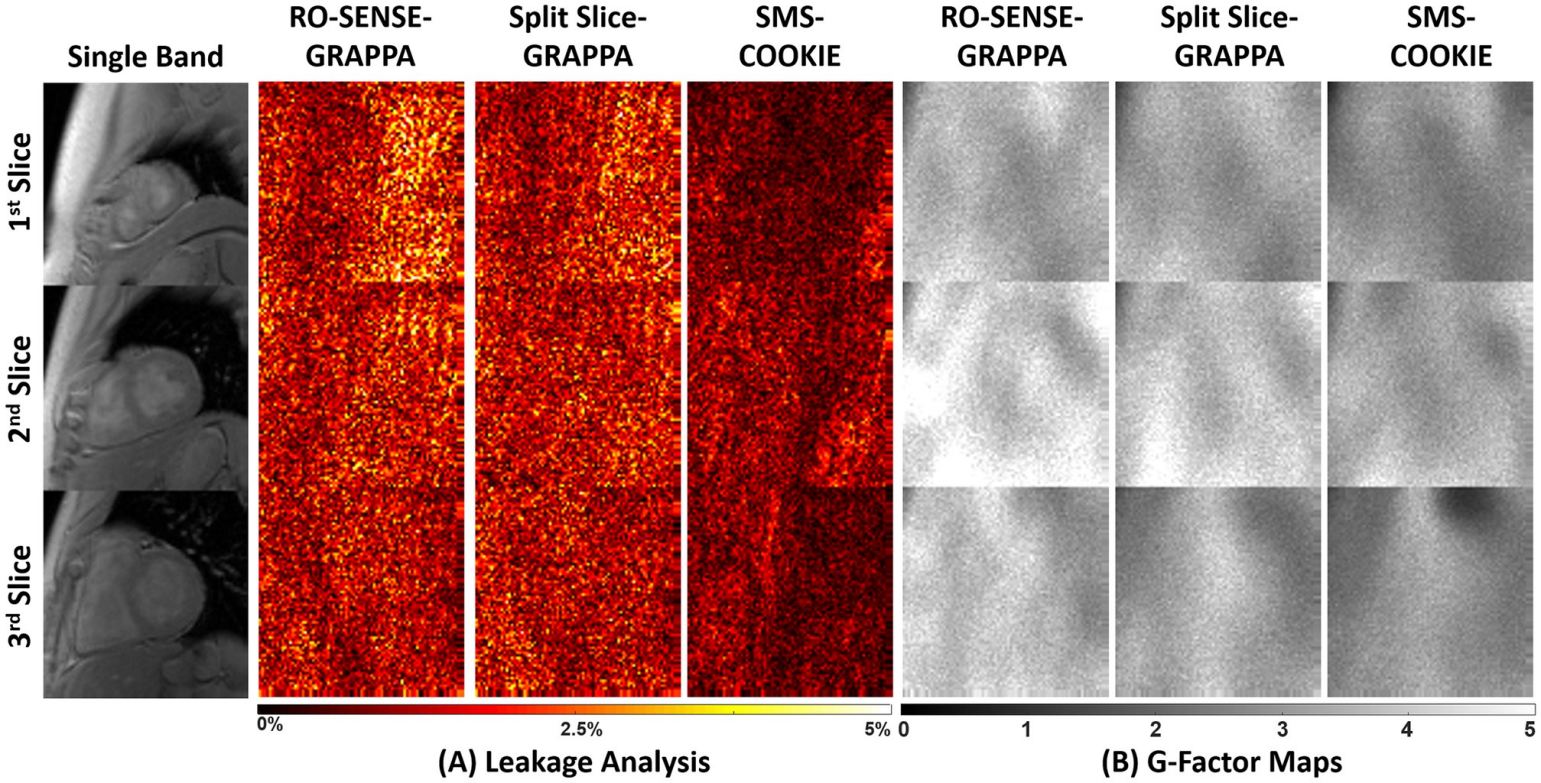
Representative leakage and g-factor maps from a retrospectively SMS-accelerated dataset. (A) Highest leakage is exhibited in RO-SENSE-GRAPPA (up to 6.5%), which is reduced using split slice-GRAPPA (up to 6.2%). The least amount of leakage is observed with SMS-COOKIE (up to 3.5%). (B) Highest g-factor values are observed in RO-SENSE-GRAPPA (mean = 3.76), corresponding to highest noise amplification, while the lowest g-factor values are shown in SMS-COOKIE (mean = 2.84). Leakage analysis and g-factor quantification require linearity in image reconstruction, therefore regularized SMS-COOKIE cannot be included in these analyses.

Fig 3 depicts representative leakage and g-factor maps. Regularized SMS-COOKIE is not included in the analysis due to its nonlinear nature. RO-SENSE-GRAPPA shows up to 6.5% leakage where split slice-GRAPPA shows slightly less with up to 6.2%. SMS-COOKIE improves upon both methods and shows only up to 3.5% leakage Fig 3(A). In terms of g-factor maps, RO-SENSE-GRAPPA shows the highest g-factor value with a mean of 3.76, whereas split slice-GRAPPA presents a lower g-factor value with 3.30. SMS-COOKIE shows the smallest g-factor value with a mean of 2.84 and provides more spatially uniform g-factors over the heart region Fig 3(B).

**Fig 4:**
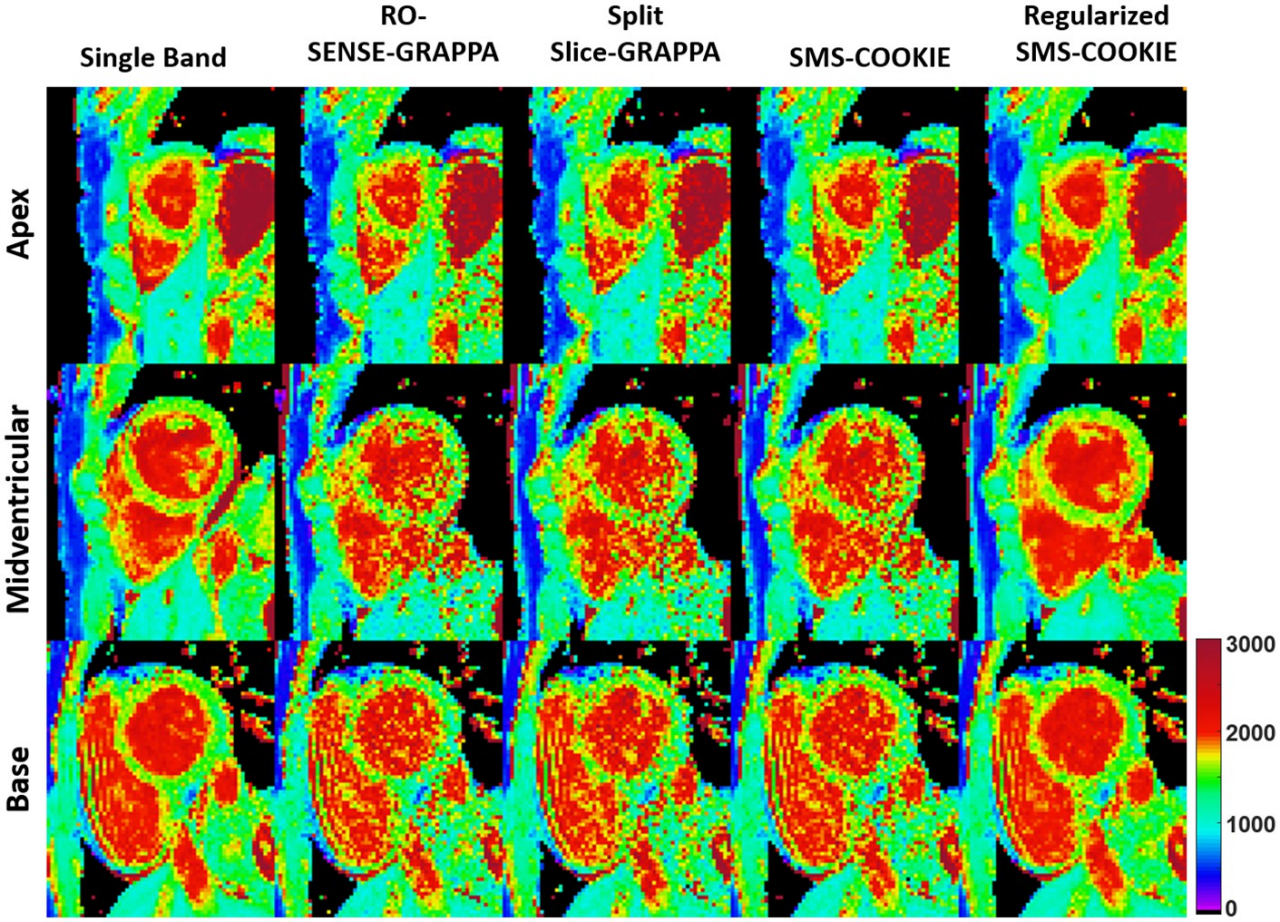
Quantitative pixel-wise tissue characterization as T_1_ maps of the three slices covering the heart in a retrospectively three-fold SMS and two-fold in-plane accelerated imaging. Single band, RO-SENSE-GRAPPA, split slice-GRAPPA, proposed SMS-COOKIE amd proposed regularized SMS-COOKIE results are shown. Regularized SMS-COOKIE exhibits closer match to single band image in terms of visual quality and shows less noise compared to other reconstruction methods.

Fig 4 shows representative pixel-wise T_1_ parameter maps of the three slices covering the heart corresponding to the apex (top), midventricular (middle) and base (bottom) from a retrospectively accelerated dataset. Single band acquisition is shown in the leftmost column as the baseline. RO-SENSE-GRAPPA and split slice-GRAPPA show similar image quality with no aliasing but visible spatial variations, and SMS-COOKIE without regularization shows modest improvement. Regularized SMS-COOKIE improves upon all methods and shows closer image quality to the single band acquisition. Furthermore, regularized SMS-COOKIE shows the least noise amplification compared to other reconstruction methods.

**Fig 5:**
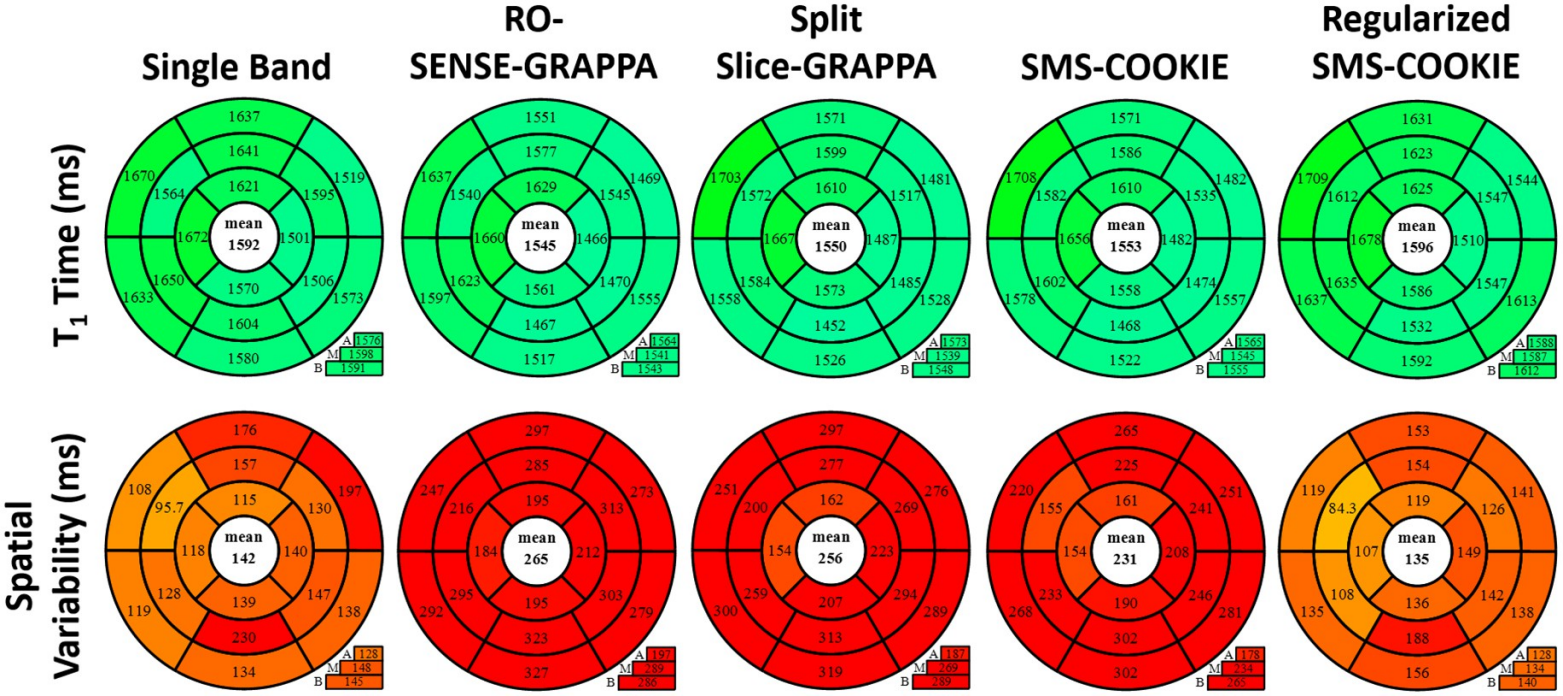
Bullseye representation of myocardial T_1_ times and T_1_ spatial variability over all subjects in retrospectively SMS accelerated study. Among non-regularized SMS methods, SMS-COOKIE shows the lowest spatial variability. When regularization is included, SMS-COOKIE further improves the spatial variability.

Bullseye representation of the quantitative evaluation of myocardial T_1_ times (ms) and spatial variabilities (ms) are depicted in Fig 5 for the retrospectively accelerated datasets. All 16 myocardial segments for single band and SMS reconstruction approaches across six subjects are shown. All reconstruction techniques yield similar T_1_ values (< 2.9% difference and *P* > 0.32). Among non-regularized reconstructions, SMS-COOKIE shows the lowest spatial variability (231 ms) followed by split slice-GRAPPA (256 ms) and RO-SENSE-GRAPPA (265 ms) whereas regularized SMS-COOKIE improved upon all and showed the least spatial variability (135 ms). All non-regularized methods show significantly different spatial variability compared to single band reference (*P* < 10^−4^) while regularized SMS-COOKIE shows no significant difference with *P* = 0.98.

### Prospectively SMS-accelerated myocardial T_1_ mapping results

**Fig 6:**
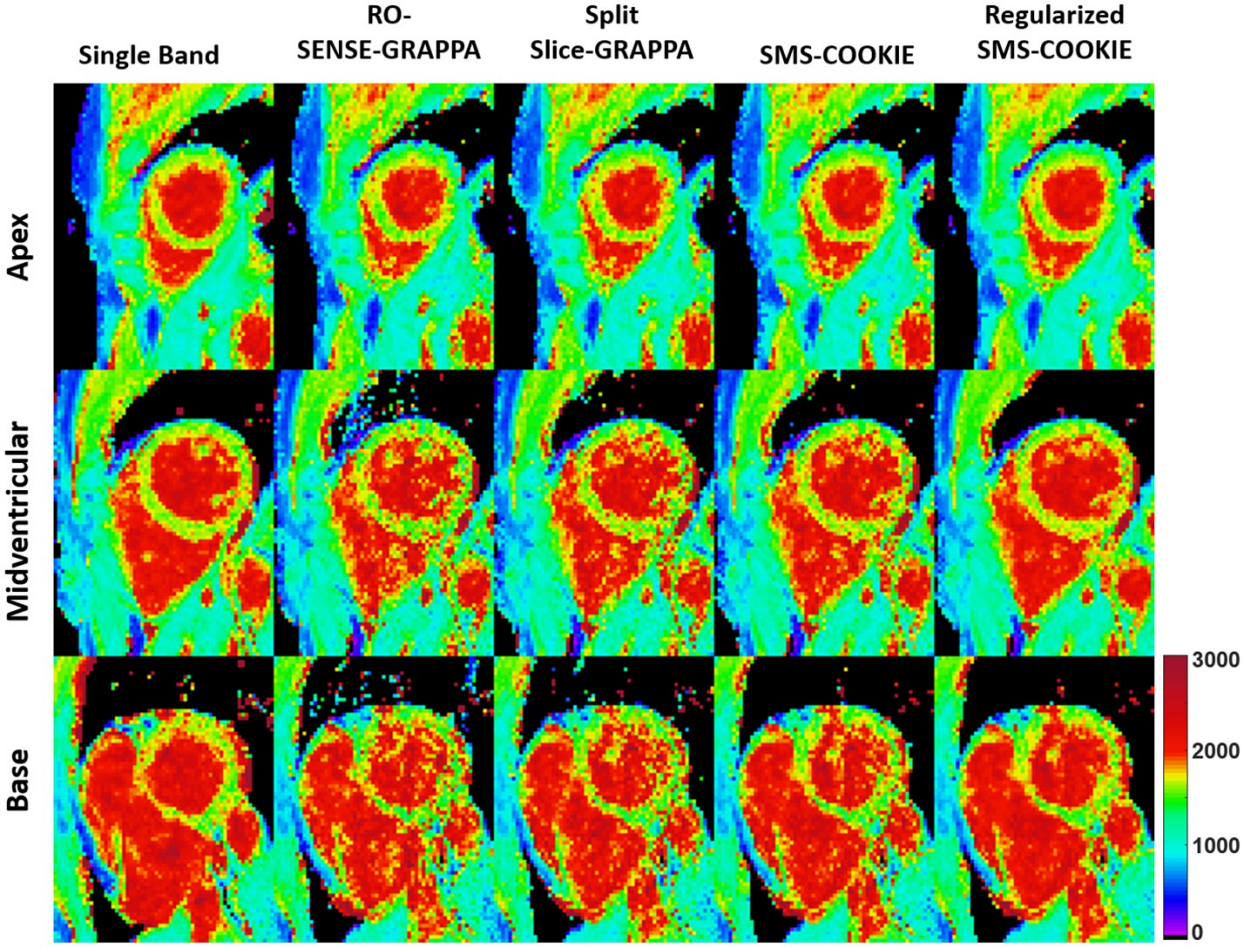
Quantitative evaluation as T_1_ maps of the three slices covering the heart in a prospectively accelerated SMS acquisition. All 4 reconstructions are shown with single band as the reference tissue characterization on the leftmost column. Regularized SMS-COOKIE s hows the closest visual quality to single band references.

The prospectively accelerated SMS acquisition with four different reconstruction approaches are shown in Fig 6, along with a separate single band acquisition in the leftmost column. Similar to the retrospectively accelerated case, SMS-COOKIE slightly improves upon both RO-SENSE-GRAPPA and split slice-GRAPPA in terms of image quality, whereas regularized SMS-COOKIE yields better visual map quality and a closer match to single band reference.

**Fig 7:**
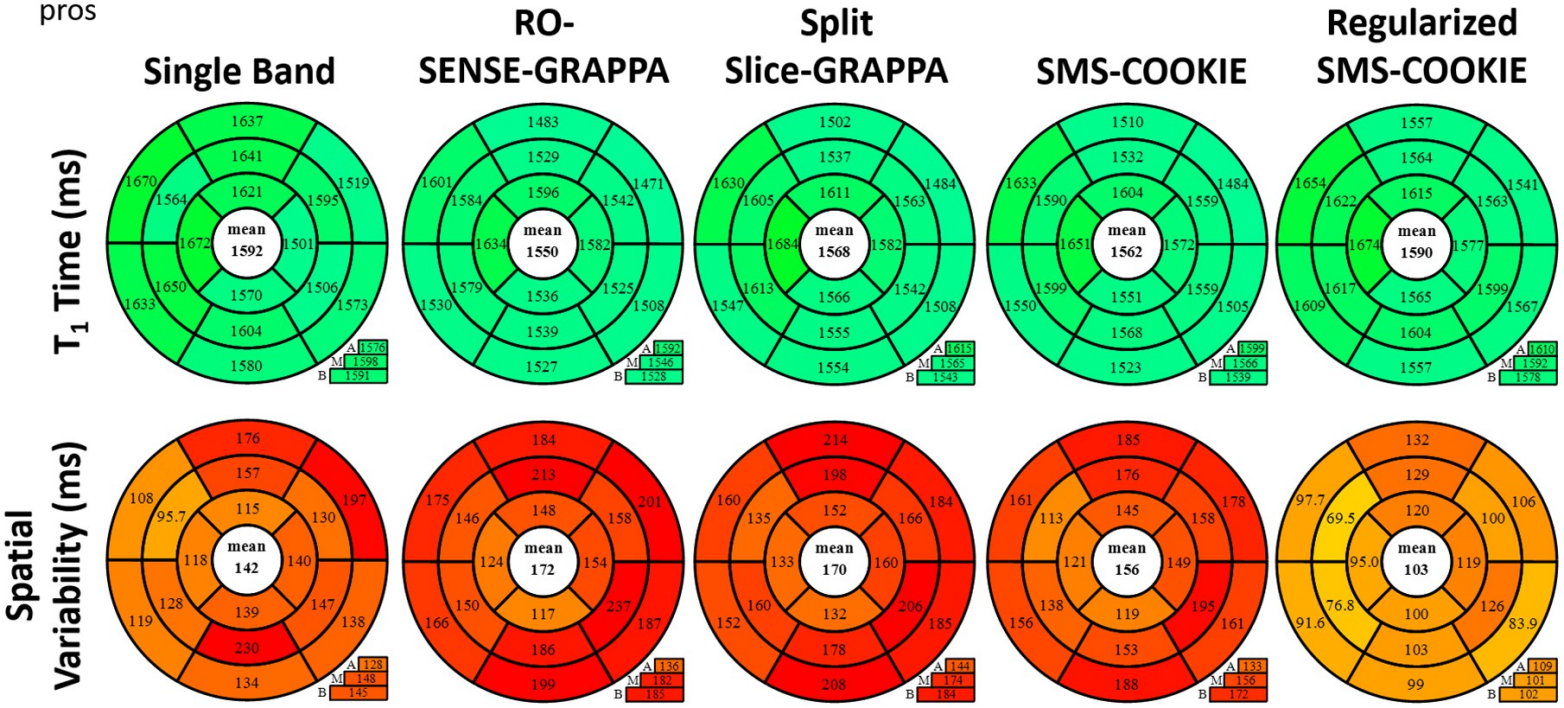
Prospectively SMS accelerated study with bullseye representation of myocardial T_1_ times and T_1_ spatial variability over all subjects. SMS-COOKIE shows the lowest spatial variability compared to non-regularized SMS reconstruction methods. Additionally, regularized SMS-COOKIE further improves the spatial variability compared to existing methods and showed less spatial variability compared to single band reference.

Fig 7 depicts the quantitative evaluation of myocardial T_1_ times (ms) and spatial variabilities (ms) using prospectively SMS-accelerated myocardial T_1_ datasets. All reconstruction techniques again yield similar T_1_ values (< 2.6% difference and *P* > 0.053). Spatial variability in the myocardium is improved using regularized SMS-COOKIE, with the least amount of spatial variability (103 ms). RO-SENSE-GRAPPA shows the highest spatial variability (172 ms), which is also significantly different than single band reference *P* < 0.04. Split slice-GRAPPA (170 ms) shows similar spatial variability to RO-SENSE-GRAPPA, while SMS-COOKIE (156 ms) shows improved spatial variability. Both methods show no significant difference compared to single band reference (*P* > 0.057). Regularized SMS-COOKIE has the lowest spatial variability (103 ms), which is in fact an improvement over single band reference (*P* < 0.002). Note that, in the prospectively SMS-encoded case, the SNR is inherently higher than the retrospective acceleration which leads to a precision improvement when using a regularized reconstruction.

### Numerical phantom results

**Fig 8:**
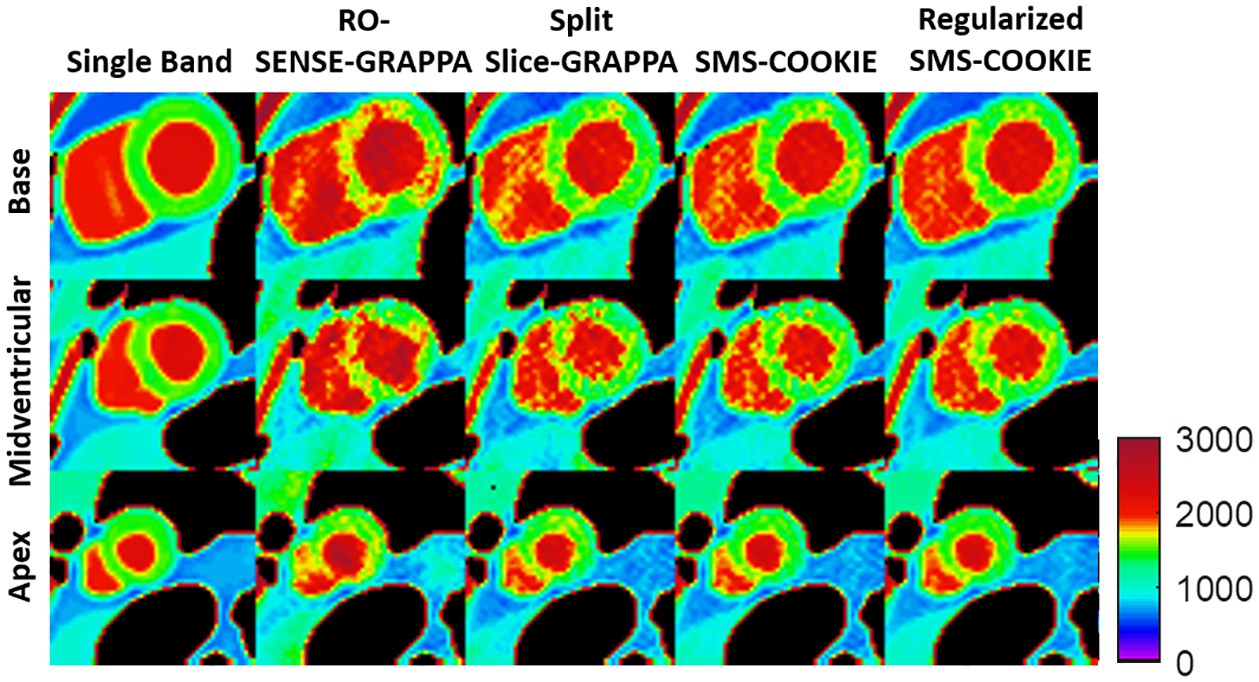
Quantitative pixel-wise tissue characterization as *T*_1_ maps of the three slices covering the heart in a simulation study with three-fold SMS and two-fold in-plane acceleration. Regularized SMS-COOKIE improves upon all methods and shows closest image quality to reference images depicted as single band images.

Fig 8 depicts representative pixel-wise *T*_1_ parameter maps of the three slices from the numerical simulations, covering the base (top), midventiruclar (middle) and apex (bottom) with 3-fold SMS and 2-fold in-plane acceleration. Reference maps are shown in the left-most column as single band images. RO-SENSE-GRAPPA suffers from aliasing artifacts which are reduced in split slice-GRAPPA but still visible in midventricular myocardium and improved by SMS-COOKIE. Regularized SMS-COOKIE shows the closest images to single band and improved upon all.

**Fig 9:**
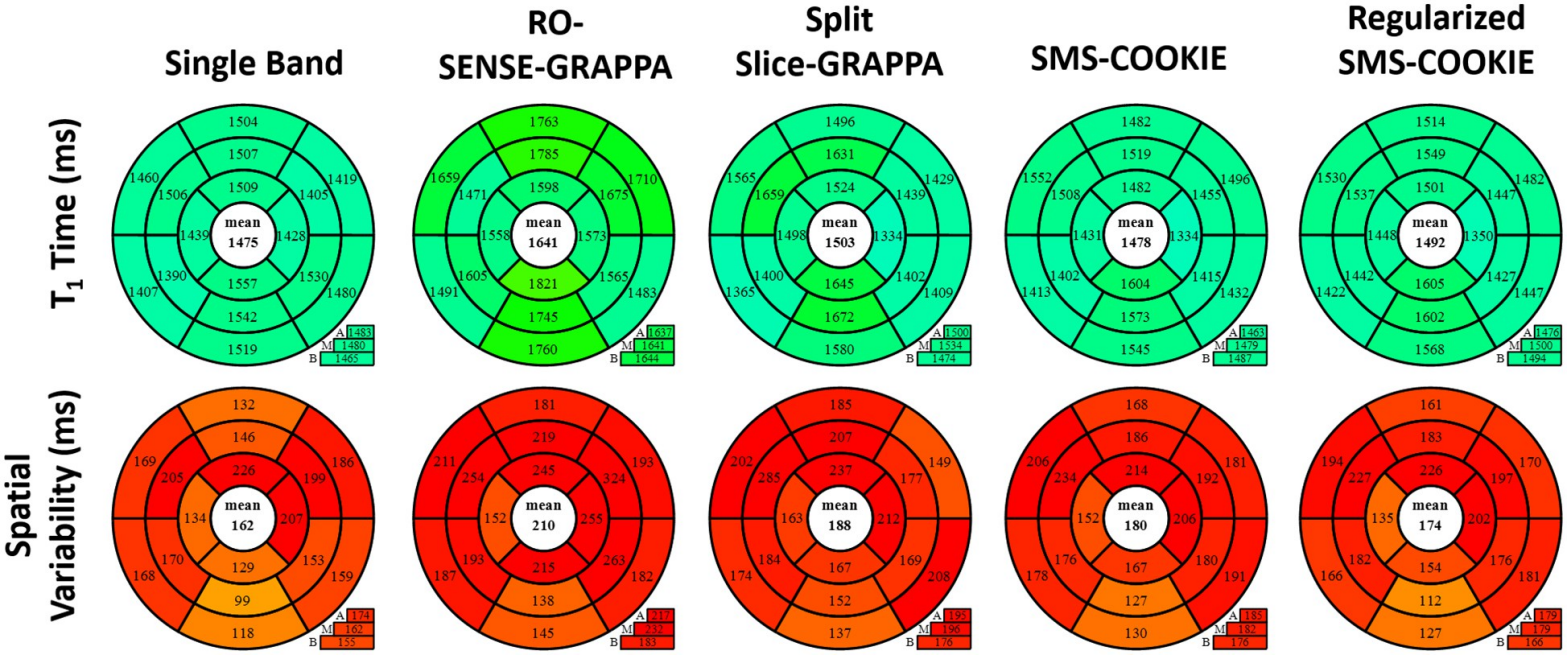
Bullseye representation of myocardial *T*_1_ times and T1 spatial variability in 3-fold SMS and 2-fold in-plane accelerated simulation study. SMS-COOKIE shows the least spatial variability among non-regularized SMS methods, which is further improved by regularized SMS-COOKIE.

Bullseye representation of the quantitative evaluation of myocardial *T*_1_ times (ms) and spatial variabilities (ms) are depicted in Fig 9. 16 segment model shows that all reconstruction techniques yield similar *T*_1_ values (< 1.9% difference and P > 0.2) except RO-SENSE-GRAPPA (< 11.3% difference and P < 7 × 10^−3^). Furthermore, regularized SMS-COOKIE shows the least spatial variability (174 ms) and improves upon all other SMS reconstruction methods (P < 10^−3^).

Table 2 shows the PSNR and SSIM metrics averaged over all slices and 15 images of each reconstruction method. Among non-regularized reconstructions, SMS-COOKIE shows the highest PSNR and SSIM, while regularized SMS-COOKIE shows the highest PSNR and SSIM among all methods. Results for additional numerical phantom experiments with 4-fold and 2-fold SMS accelerations are provided in S1 File.

**Table 2:** Average PSNR and SSIM metrics over 15 images and all three slices for simulation study at 3-fold SMS and 2-fold in-plane acceleration. Regularized SMS-COOKIE shows the highest PSNR and SSIM performance compared to non-regularized methods.

Method	PSNR	SSIM
RO-SENSE-GRAPPA	24.5 ± 3.1	87.3 ± 4.3
Split Slice-GRAPPA	27.0 ± 2.5	88.7 ± 4.3
SMS-COOKIE	27.7 ± 2.4	89.5 ± 4.4
Regularized SMS-COOKIE	33.7 ± 0.3	92.1 ± 4.8

## Discussion

In this study, we proposed a new regularized reconstruction method called SMS-COOKIE for SMS imaging and applied it to myocardial T_1_ mapping. Both non-regularized and regularized versions of SMS-COOKIE yielded good agreement with single band T_1_ times. Non-regularized SMS-COOKIE provided lower spatial variability than commonly used leakage-blocking SMS reconstruction techniques, split slice-GRAPPA and RO-SENSE-GRAPPA. Furthermore, regularized SMS-COOKIE reduced noise further and achieved significantly lower spatial variability of myocardial T_1_ times than split slice-GRAPPA.

SMS imaging combined with in-plane acceleration is prone to noise amplification despite the CAIPIRINHA phase shifts for controlled aliasing. GRAPPA-type methods have been shown to lead to successful reconstruction for myocardial T_1_ mapping with lower leakage compared with SENSE-type reconstruction [23]. Even with its desirable leakage properties, split slice-GRAPPA suffers from noise amplification, and GRAPPA-type reconstructions do not allow further direct regularization for noise reduction. Meanwhile SPIRiT-type reconstruction facilitates noise reduction via incorporation of regularization in its objective function. SMS-COOKIE uses advantages of both GRAPPA-type and SPIRiT-type reconstructions to improve image quality.

In parallel imaging, g-factor maps, which depend on coil geometry and acceleration rate, describe the spatially varying noise amplification [47]. Although, g-factor analysis is performed for RO-SENSE-GRAPPA, split slice-GRAPPA and proposed SMS-COOKIE, regularized SMS-COOKIE is non-linear in nature, and such a g-factor quantification cannot be performed [39]. In addition to g-factor maps, interslice leakage, as a measure of cross-talk across simultaneously excited slices can be quantified [28, 29]. Similar to g-factor quantification, leakage analysis depends on the linearity of the image reconstruction. Therefore, leakage analysis was not performed for the non-linear reconstructions in regularized SMS-COOKIE. Thus, we have additionally used the spatial variability in T_1_ maps as a surrogate for noise amplification. Spatial variation caused by physiology is expected to be low in this cohort of healthy people as previously reported [23]. Hence, most of the spatial variability is commonly attributed to noise effects [10, 23].

The empirically tuned thresholding parameter for the LLR regularization from retrospective study is divided by three (the SMS acceleration factor) in prospectively accelerated SMS-encoding due to the inherently higher SNR. In the retrospective acceleration study, data from all three slices are summed to simulate the SMS encoding, yielding combined noise whose variance is higher by a factor of the SMS-acceleration due to the combination of the independent noise from the individual slices.

The choice of split slice-GRAPPA as an additional constraint in SMS-COOKIE formulation does not merely serve as a different starting point for optimization, but sets a baseline interpolation with leakage-blocking properties and serves as a key part of the objective function in Eq 3. Additionally, split slice-GRAPPA [29] was utilized in this study instead of slice-GRAPPA [31], since it is more commonly used in practice due to its favorable leakage properties, yet it leads to higher noise amplification, further making noise improvement as in SMS-COOKIE critical.

## Conclusion

In this work, we proposed and evaluated an SMS imaging reconstruction technique that combines the advantages of GRAPPA and SPIRiT type k-space interpolation. The proposed SMS-COOKIE reconstruction showed improved spatial variability compared to split slice-GRAPPA and SMS-SPIRiT in myocardial T_1_ mapping. Additionally, this method allowed for the incorporation of regularizers for further reduction of reconstruction noise.

## Supporting information

S1 File The supporting information for parameter tuning of weight terms and additional numerical phantom experiments. (PDF)
