# Supplementary material for: Improved Simultaneous Multi-slice imaging with Composition of k-space Interpolations (SMS-COOKIE) for myocardial T1 mapping
Source: PLoS One. 2023 Jul 21;18(7):e0283972. doi: 10.1371/journal.pone.0283972 (PMC10361528; doi:10.1371/journal.pone.0283972)
Supplement: S1 File — (PDF) [file pone.0283972.s001.pdf]

Supporting Information

SMS-COOKIE Parameter Tuning

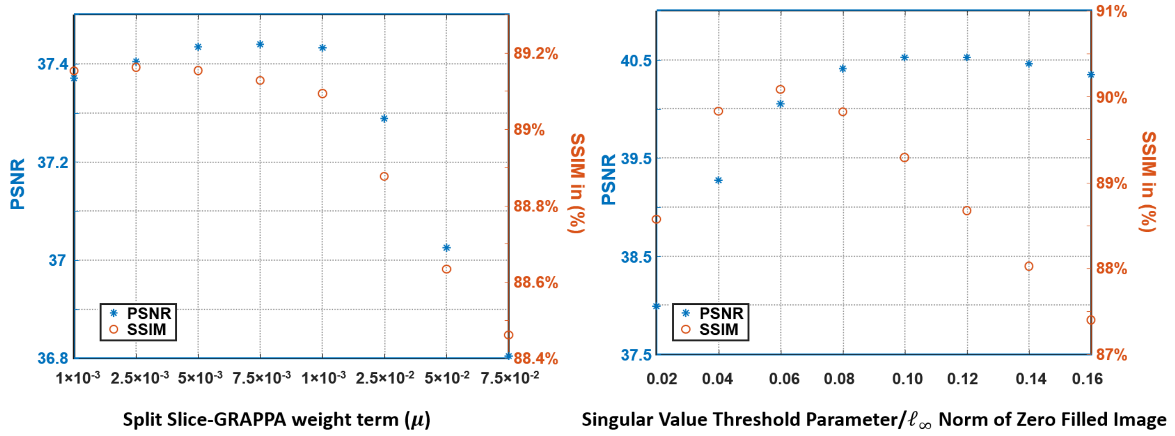

Supporting Information Figure S1: The parameter tuning for split slice-GRAPPA weight term (μ) and singular value threshold parameter for regularization. The split slice-GRAPPA weight term (μ) in Equation 3 was empirically tuned in a subject to 7.5×10^{-3}. Additionally, the singular value thresholding parameter (σ_i/ρ) was empirically tuned to 0.08 times of the ℓ_∞ norm of the SENSE-1 images for all slices in regularized SMS-COOKIE using a separate subject.

Additional Numerical Phantom Experiments

Additional experiments with lower SMS factor but increased in-plane acceleration (2-fold SMS and 3-fold in-plane acceleration) and higher SMS factor without in-plane acceleration (4-fold SMS acceleration) were conducted. **Supporting Information Figure S2** shows the representative pixel-wise T_1 parameter maps of the four slices from the simulation study covering the base (top), midventricular (2nd row), apical (3rd row) and apex (bottom). RO-SENSE-GRAPPA suffer from noise amplification while split slice-GRAPPA shows residual artifacts (midventricular and apical

slices). SMS-COOKIE improves upon all non-regularized methods while regularized SMS-COOKIE shows the closest image quality to single band (reference) maps.

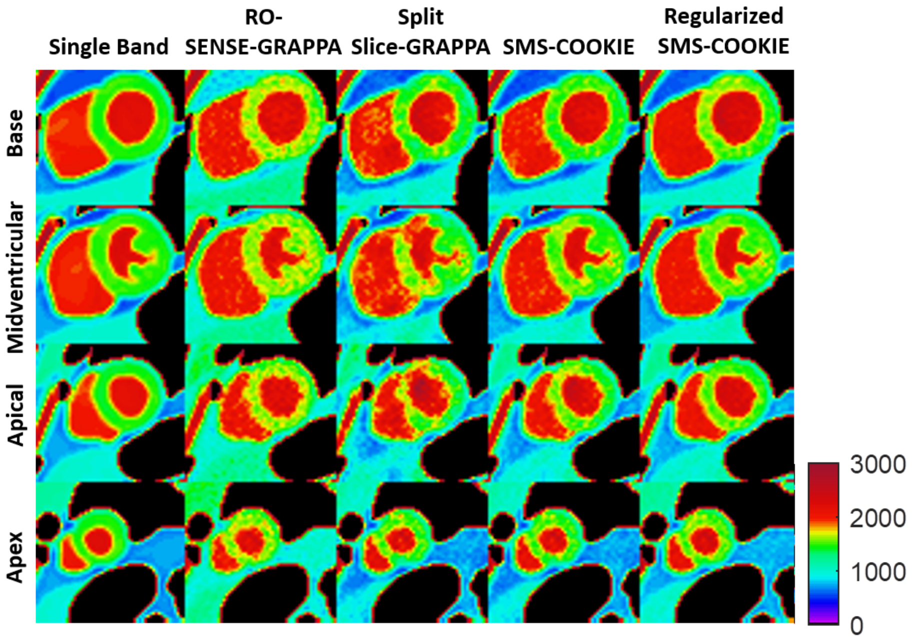

Supporting Information Figure S2: Quantitative pixel-wise tissue characterization as T_1 maps of the four slices covering the heart in a simulation study with four-fold SMS acceleration. Regularized SMS-COOKIE improves upon all methods and shows closest image quality to reference images depicted as single band images.

Bullseye representation of the quantitative evaluation of myocardial T_1 times (ms) and spatial variabilities (ms) are depicted in **Supporting Information Figure S3** for the simulation study at 4-fold SMS acceleration. This time a 17 segment model is used that shows all reconstruction techniques yield similar T_1 values (<5.5% difference and $P > 0.1$) except RO-SENSE-GRAPPA (<9.0% difference and $P < 2 \cdot 10^{-3}$). Mean values of the T_1 times and spatial variabilities of all 17 segments are depicted in the small black boxes. Regularized SMS-COOKIE shows the least spatial

variability (183 ms) and improved upon all methods where all show significantly different spatial variability compared to single band references (P < 0.02).

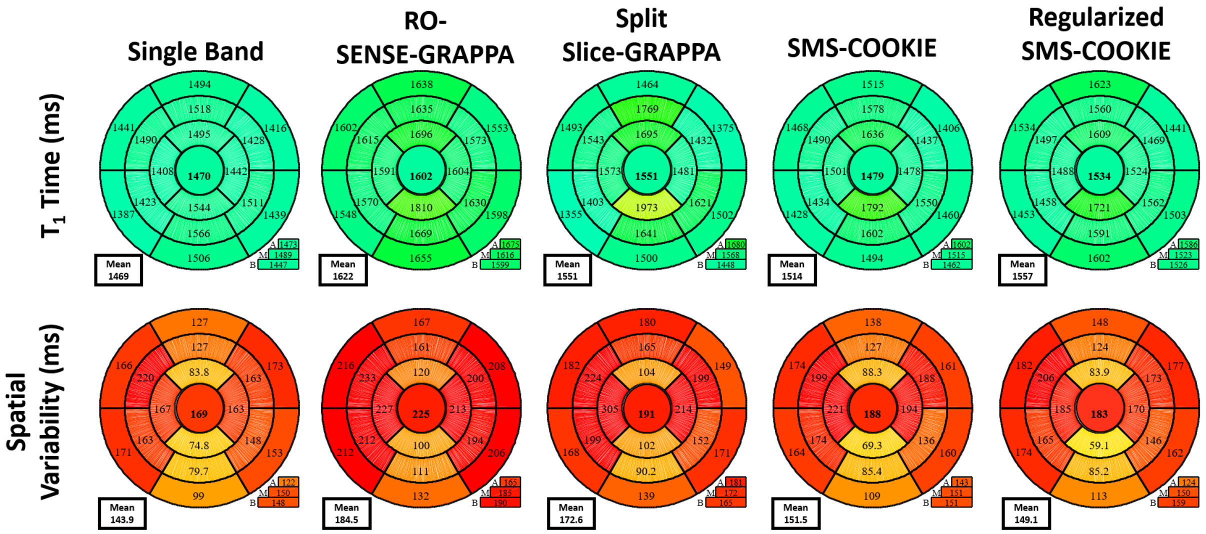

Supporting Information Figure S3: Bullseye representation of myocardial T_1 times and T_1 spatial variability in 4-fold SMS accelerated simulation study. Among non-regularized SMS methods, SMS-COOKIE shows the lowest spatial variability and improved by regularized SMS-COOKIE.

Additionally, PSNR and SSIM metrics averaged over all four slices and 15 images of each reconstruction method are depicted in **Supporting Information Table S1** where regularized SMS-COOKIE shows the highest PSNR and SSIM among all improved upon closes competitor non-regularized SMS-COOKIE by 8.5% ins PSNR and 5.8% in SSIM.

**Method**	**PSNR**	**SSIM**
**RO-SENSE-GRAPPA**	27.2±7.4	83.9±18.4
**Split Slice-GRAPPA**	29.9±9.8	84.1±18.8
**SMS-COOKIE**	30.9±10.8	85.3±18.2
**Regularized SMS-COOKIE**	33.8±9.0	90.6±11.8

Supporting Information Table S1: Average PSNR and SSIM metrics over 15 images and all four slices. Regularized SMS-COOKIE shows the highest PSNR and SSIM performance compared to non-regularized methods.

Lastly, **Supporting Information Figure S4** depicts the representative pixel-wise T_1 parameter maps of the two slices from the simulation study covering the base (top) and midventiruclar (bottom) with 2-fold SMS and 3-fold in-plane acceleration. RO-SENSE-GRAPPA and split slice-GRAPPA suffer from noise amplification and aliasing artifacts whereas SMS-COOKIE improves upon these and regularized SMS-COOKIE shows the closest image quality to single band (reference) maps.

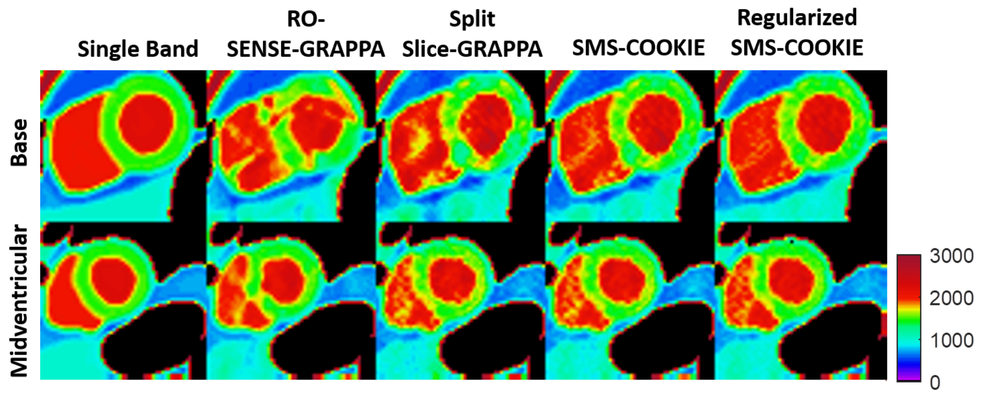

Supporting Information Figure S4: Quantitative pixel-wise tissue characterization as T1 maps of the two slices in a simulation study with two-fold SMS and three-fold in-plane acceleration. Regularized SMS-COOKIE improves upon all methods and shows closest image quality to reference images depicted as single band images.

Bullseye representation of the quantitative evaluation of myocardial T_1 times (ms) and spatial variabilities (ms) are depicted in **Supporting Information Figure S5** for the simulation study at 2-fold SMS and 3-fold in-place acceleration by keeping 24 ACS lines. This time a 12 segment model is used (the apex segments are left empty) where all reconstruction techniques yield similar T_1 values (<1.0% difference and $P > 0.2$). Regularized SMS-COOKIE shows the least spatial variability (123 ms) and improved upon all methods where only split slice-GRAPPA show significantly different spatial variability compared to single band references ($P < 0.03$) while both non-regularized and regularized SMS-COOKIE do not show ($P > 0.05$).

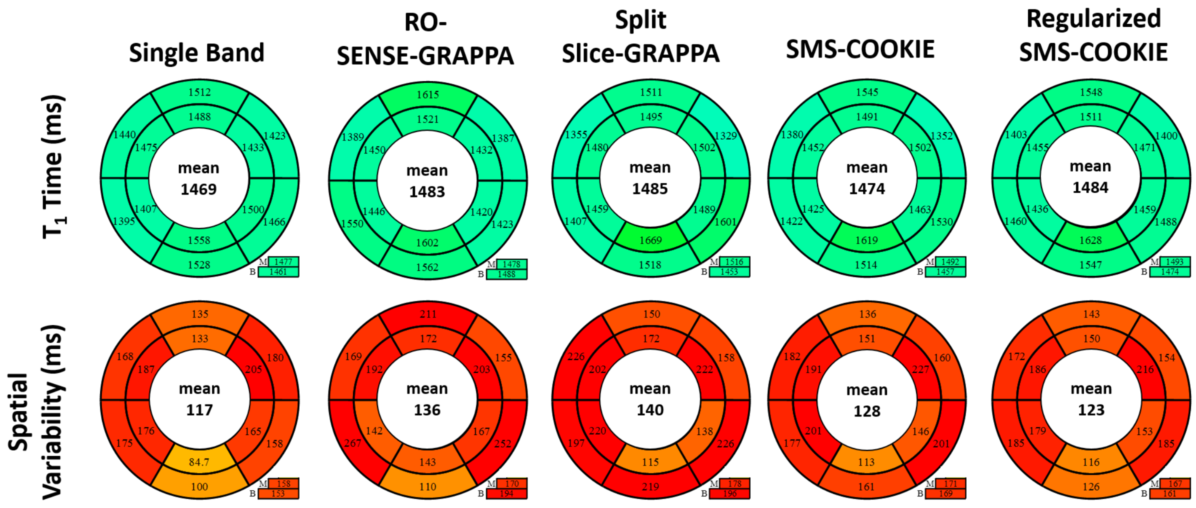

Supporting Information Figure S5: Bullseye representation of myocardial T_1 times and T_1 spatial variability over all subjects in 2-fold SMS and 3-fold in-plane accelerated simulation study. Among non-regularized SMS methods, SMS-COOKIE shows the lowest spatial variability and improved by regularized SMS-COOKIE.

Additionally, PSNR and SSIM metrics averaged over all two slices and 15 images of each reconstruction method are depicted in **Supporting Information Table S2** where regularized SMS-COOKIE shows the highest PSNR and SSIM among all improved upon closes competitor non-regularized SMS-COOKIE by 5.0% in PSNR and 5.5% in SSIM.

**Method**	**PSNR**	**SSIM**
**RO-SENSE-GRAPPA**	24.5±5.7	81.8±14.3
**Split Slice-GRAPPA**	29.4±9.6	83.2±16.6
**SMS-COOKIE**	29.8±10.0	84.9±15.4
**Regularized SMS-COOKIE**	31.4±11.9	89.9±11.0

Supporting Information Table S2: Average PSNR and SSIM metrics over 15 images and all four slices. Regularized SMS-COOKIE shows the highest PSNR and SSIM performance compared to non-regularized methods.
